# Production Performance and Properties of Eggs from Hens Fed Diets Differing in Corn Grain Hardness, Vitamin A Supplementation Level, and Mineral Form

**DOI:** 10.3390/foods15040692

**Published:** 2026-02-13

**Authors:** Kristina Kljak, Dora Zurak, Goran Kiš, Zlatko Janječić, Dalibor Bedeković, Helga Medić, Vasil Pirgozliev, Nives Marušić Radovčić

**Affiliations:** 1 Faculty of Agriculture, University of Zagreb, Svetošimunska 25, 10000 Zagreb, Croatia; kkljak@agr.hr (K.K.); dzurak@agr.hr (D.Z.); kis@agr.hr (G.K.); zjanjecic@agr.hr (Z.J.); dbedekovic@agr.hr (D.B.); 2 Faculty of Food Technology and Biotechnology, University of Zagreb, Pierottijeva 6, 10000 Zagreb, Croatia; hmedic@pbf.hr; 3 National Institute of Poultry Husbandry, Harper Adams University, Newport TF10 8NB, UK; vpirgozliev@harper-adams.ac.uk

**Keywords:** laying hen, egg yolk, retinol, tocols, fatty acid profile, egg quality, oxidative stability

## Abstract

This study evaluated how corn grain hardness, vitamin A supplementation level, and trace mineral form influence production performance and egg properties in laying hens. In a 2 × 3 × 2 factorial design, 252 Lohmann Brown hens received diets containing soft- or hard-type corn hybrids; 5000, 10,000, or 20,000 IU/kg of vitamin A; and inorganic or organic trace minerals for 63 days. Hard-type corn increased daily egg mass, improved feed conversion ratio, and produced eggs with higher MUFAs and SFAs but lower PUFAs and n-3, resulting in a less favorable n6/n3 ratio, while also increasing susceptibility to Fe-induced lipid oxidation despite lower PUFAs. Increasing dietary vitamin A to 10,000–20,000 IU/kg increased egg weight and shell strength, linearly increased yolk retinol, and decreased tocols, with 20,000 IU/kg markedly increasing Fe-induced MDA formation without major changes in PUFAs. Trace mineral form had minor effects on performance and fatty acid profile. Overall, modest changes in laying hen diet, such as corn hybrid, vitamin A supplementation level, and trace mineral form, significantly modulated egg nutritional composition and oxidative stability. A high dietary vitamin A level may compromise the oxidative resilience of enriched eggs, while interactions of trace mineral form with corn hybrid and vitamin A suggest its potential modulatory role in lipid oxidation pathways.

## 1. Introduction

The nutritional composition of table eggs can be manipulated by changing the diet of laying hens, in particular by the inclusion of fat-soluble vitamins, carotenoids, trace minerals, and fatty acids. Such dietary enrichment could be strategically used to improve the nutritional quality and functional value of eggs for human consumption [1]. Eggs are an important source of retinol (vitamin A), tocols (vitamin E), and polyunsaturated fatty acids (PUFAs) in the human diet [2]. The edible part of the egg (100 g) contains 160 µg of vitamin A, 1050 µg of vitamin E, and 1.65 g of PUFAs, corresponding to 15–20, 8, and 18–36% of the daily recommendations for vitamin A, vitamin E, and eicosapentaenoic acid (EPA) plus docosahexaenoic acid (DHA), respectively [3,4,5]. Vitamin A and vitamin E play an essential role in maintaining immune function and antioxidant protection [6], while PUFAs contribute to cardiovascular and cognitive health through anti-inflammatory mechanisms [7]. However, the enrichment of eggs with these compounds, especially PUFAs, can increase their susceptibility to oxidative degradation [8], which has a negative impact on the nutritional value and shelf life of eggs.

In addition to the effects on human health, these compounds also play an important role in the physiological health and productivity of laying hens. Vitamin A supports epithelial integrity, immune function, and reproductive performance, with supplementation (6000–8000 IU/kg) improving antioxidant enzyme activity (SOD, CAT, and GPx), fertility, hatchability, and immune markers in aged hens and their offspring [9]. Vitamin E improves cellular antioxidant protection [10], and PUFAs improve bone health, fertility, and immune response [11]. Consequently, these compounds influence the production performance of laying hens. For example, higher vitamin supplementation levels improve laying performance and egg quality [12], while PUFAs can influence egg weight [13]. However, it must be taken into account that some compounds may compete for absorption, resulting in lower bioavailability. For example, competing absorption between vitamin A and carotenoids can affect their bioavailability and deposition in the yolk [14]. Furthermore, trace minerals such as zinc, selenium, copper, and manganese contribute to eggshell formation, enzyme function, and antioxidant protection [15].

The efficiency of nutrient deposition into eggs and their biological effects depend not only on the level of dietary intake, which is the most common form of dietary manipulation, but also on the properties of the feeds and additives in the laying hen diet. Corn grain is a major energy source in poultry diets; however, nutrient utilization depends on grain properties, which are genotype-dependent. According to in vitro digestibility measurements, zein content as well as the size and shape of the starch grains influence the starch digestibility rate [16], which in turn affects nutrient utilization and production performance [17]. Zein content and starch granule properties are related to grain vitreousness and the ratio of vitreous to floury endosperm, which affects grain hardness [18]. Grain hardness also plays an important role in the deposition of carotenoids in the yolk [19].

Considering the effects of hybrids on the utilization of starch and carotenoids, it is possible that hybrids also affect other corn nutrients, such as tocols and fatty acids. A comparative evaluation of 103 commercial hybrids by Gunjević et al. [20] revealed considerable differences in both total and bioaccessible tocol content (tocopherols and tocotrienols), suggesting that selection of hybrids with higher bioaccessible tocol content could improve vitamin E supply to laying hens. Additionally, Goffman and Böhme [21] reported differences in the fatty acid profiles of corn hybrids, particularly for palmitic, oleic, and linoleic acids. Also, they found moderate correlations between vitamin E compounds and PUFAs. This suggests that hybrid-dependent differences in the composition of grain oil could lead to altered fatty acid deposition in yolks and the nutritional quality of eggs. Furthermore, the fatty acid profile of corn hybrids is related to grain hardness. The vitreous endosperm is characterized by a dense protein–starch matrix in which the lipids are structurally more embedded than in the floury endosperm, which affects both the total lipid content and the profile of fatty acids, especially unsaturated ones such as oleic and linoleic acids [22].

Trace elements can be added to the diet of laying hens in inorganic (oxides, sulfates) or organic (chelated, protein-bound) form. Studies have shown that the latter form is more bioavailable, so it is supplemented in smaller quantities in the laying hen diet [23]. They also provide stronger antioxidant support with lower supplementation levels compared to the inorganic form [15]. As a result, organic forms improve the production performance of laying hens and egg quality [24].

The dietary- and ingredient-dependent properties of laying hen diets are usually studied by considering one or two dietary components, and relatively few have investigated the interaction of multiple ingredients in a single trial. The interaction between corn hybrid characteristics, dietary vitamin A level, and trace mineral form may affect laying hen production performance and egg properties, and their combined inclusion may have complementary effects. For example, simultaneous enrichment of eggs with vitamin E, carotenoids, selenium, and DHA may enhance the oxidative stability of yolk PUFAs during storage and heat treatment, increase nutritional value through higher availability of fat-soluble compounds such as vitamin E and carotenoids, and reduce the risk of oxidation-related off-flavors [25]. Therefore, the aim of the present study was to investigate the effects of these three nutrient factors and their interactions on the production performance of laying hens and the nutritional quality of the eggs they produce.

## 2. Materials and Methods

All animal procedures complied with Croatian animal welfare legislation (Animal Protection Act, OG 102/17 and 32/19; the Regulation on the Protection of Animals Used for Scientific Purposes, OG 55/13, 39/17, and 116/19), which correspond to the relevant European requirements for the use of animals in research. Ethical approval was granted by the Committee for the protection of animals used in scientific research within the Ministry of Agriculture of the Republic of Croatia (EP 349/2022).

### 2.1. Grain Production

Two commercial corn hybrids were chosen from an initial set of 103 commercial hybrids on the basis of their physicochemical characteristics, carotenoid bioaccessibility, and performance in a laying hen trial [26,27]. Both hybrids were of the dent type but differed in grain hardness [19]. Accordingly, the hybrid with lower grain hardness was designated as the soft-type hybrid, whereas the hybrid with higher grain hardness was designated as the hard-type hybrid throughout the present study. Seed for both hybrids was sourced from a commercial supplier.

The hybrids were cultivated during the 2022 growing season in the same experimental field in central Croatia (near Zagreb). Each hybrid was planted on a plot measuring 70 m × 50 m under identical agroclimatic conditions. Seeding density followed the seed company recommendations, and the crop was managed under an intensive production system. At maturity, maize was mechanically harvested and subsequently dried at 85 °C to a target moisture of approximately 12%. After drying, grain from each hybrid was stored in bags until it was used for the preparation of the laying hen diets.

### 2.2. Hens, Experimental Design, and Treatment Diets

The experiment included 252 Lohmann Brown laying hens (18 weeks of age). Hens were housed three per cage in a total of 84 enriched cages compliant with Council Directive 1999/74/EC, located in the experimental poultry facility of the University of Zagreb Faculty of Agriculture. The cages were arranged in four free-standing laying batteries, providing 1269 cm^2^ per hen. The height of the cage was 60 cm, and the minimum distance from the bottom to the top was 44 cm. Each cage was equipped with an external front feeder (≥12 cm per bird), two nipple drinkers, a perch (≥15 cm per hen), and a claw-shortening device. Eggs were collected in a wired egg cradle positioned parallel to and below the feeder. Environmental conditions were automatically regulated; temperature sensors distributed throughout the house enabled continuous monitoring and ventilation adjustment. The room temperature and relative humidity were maintained at 18 ± 2 °C and 55–65%, respectively, throughout the experiment. The light period consisted of 16 h of light per day, while diet and water were provided *ad libitum* to the laying hens. Artificial light was the only light source in the experimental poultry house, provided by soft-white, fluorescent bulbs mounted on the walls to ensure a light intensity of 15 lux.

Experimental diets were formulated to meet the recommended nutrient requirements of commercial Lohmann Brown laying hens in the initial stage of egg production (19 to approximately 50 weeks of age) according to the Lohmann Breeders GmbH [28] guidelines. The basal mixture contained all ingredients except corn grains, vitamin A, and minerals, and was mixed from a single batch of ingredients to reduce differences in nutrient composition. Immediately before the start of the feeding trial, the grains of both hybrids were transported to a feed mill near Zagreb, Croatia, and ground through a 6 mm sieve. Each corn hybrid was then assigned to one of the three vitamin A levels (5000, 10,000, and 20,000 IU/kg; ROVIMIX A1000, DSM-Firmenich AG’s, Kaiseraugst, Switzerland), combined with inorganic or organic trace minerals and mixed with the basal mixture, yielding a total of 12 dietary treatments. Organic trace minerals were supplied by Bioplex (Cu 12%, Fe 15%, Mn 20%, and Zn 20%; amino acid- and peptide-complexed; Alltech, Nicholasville, KY, USA) and Sel-Plex (Se 0.30%, selenium-enriched yeast; Alltech, Nicholasville, KY, USA). Inorganic trace minerals were provided as copper sulfate, ferrous sulfate, manganese oxide, zinc oxide, and sodium selenite. The ingredient composition of diets and premixes is shown in Supplementary Tables S1A and S1B, respectively. Fatty acid profile and the content of tocols in experimental diets are given in Table 1 and Table 2, respectively.

Prior to the feeding trial, the laying hens were fed a white corn-based diet with the same calculated ingredient composition as the experimental diet for 4 weeks to reach the peak of egg production. Thereafter, laying hens were randomly assigned to one of 12 dietary treatments in a 2 × 3 × 2 factorial design. The experimental design included three factors: corn hybrid (two commercial hybrids; soft- and hard-type), vitamin A supplementation level (5000, 10,000, or 20,000 IU/kg), and trace mineral form (inorganic or organic). Each dietary treatment was replicated in seven cages. The trial lasted 63 days and comprised an adaptation phase (20 days) followed by a sampling phase (43 days). During the sampling phase, egg number and egg weight were recorded daily, whereas feed intake was measured weekly using a precision balance (JL1502-G, Mettler-Toledo, Greifensee, Switzerland). These data were used to calculate performance parameters of laying hens.

From day 21 onward, eggs were collected weekly for the determination of egg quality traits and content of tocols and retinol. Eggs collected between days 30 and 35 from the beginning of the experimental trial were used for oxidative stability assessment, while eggs collected on days 38 and 39 were used for the analysis of yolk fatty acid profile. In total, 147 eggs were collected for analysis of egg quality and for tocol and retinol content, 21 eggs for fatty acid profile analysis, and 105 eggs for oxidative stability analyses per dietary treatment. For analyses requiring yolk samples, eggs were broken immediately prior to analysis, yolks were separated from albumen, and dried on a paper napkin. For tocol, retinol, and fatty acid analysis, three yolks from each cage were pooled, resulting in seven composite samples per treatment per week. Eggs were analyzed as soon as possible after collection and, when required, stored at 4 °C. Egg quality assessment was performed within 24 h of egg collection.

### 2.3. Analysis of Egg Quality and Color

The height and width of the eggs were measured with a digital caliper (Digital Caliper 150 mm, Alpha Tools, Mannheim, Germany) and used to calculate the shape index as the ratio of width to height multiplied by 100. Egg surface area (cm^2^) was calculated using the equation Ps = 4.835 × W^0.662^, where *W* represents egg weight (g) [29]. Eggshell color was measured at the large end of each egg using the Commission Internationale de l’Eclairage (CIE) color scale with a colorimeter [model CM-700d, Minolta, Osaka, Japan; lightness (L*), redness (a*), and yellowness (b*)]. The CIE L*, a*, and b* coordinates describe color attributes as follows: L* indicates lightness (0 = black; 100 = white), a* represents redness (−a = green, a = red), and b* represents yellowness (−b = blue, b = yellow), respectively.

Whole eggs were subsequently analyzed using a Digital Egg Tester (DET 6000, Nabel, Kyoto, Japan) to determine shell strength. After breaking the eggs, the albumen and yolk were transferred onto the instrument plate, and the albumen height, Haugh units, and yolk according to the Yolk Color Fan (YCF) were measured. The yolk was then separated from the albumen, and the weight of each was measured. Eggshells were rinsed, air-dried at room temperature for 24 h, weighed, and the shell thickness at the egg’s equatorial area was measured using the same instrument.

Separated egg yolks were dried on a paper napkin, and the yolks of eggs from the same cage were combined and mixed using a spatula. The combined yolks from the same cage were used to determine the L*, a*, and b* values with a colorimeter (model CR-410, Minolta Co., Ltd., Osaka, Japan). The instrument was calibrated daily against a white standard plate with the properties Y = 94.5, x = 0.3158, and y = 0.3323.

### 2.4. Analysis of Tocols and Retinol Content in Egg Yolk

Quantification of tocols and retinol from egg yolks collected from day 21 to the end of the experiment was performed using the reversed-phase HPLC method following the extraction according to Surai et al. [30], using retinyl acetate and DL-α-tocopherol acetate as internal standards. Yolk samples (200 mg) were homogenized in 2 mL of a 1:1 (*v*/*v*) mixture of 5% sodium chloride solution and ethanol. Subsequently, 3 mL of hexane was added, and the mixture was further homogenized for 3 min. Following centrifugation (1200× *g*, 5 min), the upper organic phase was transferred to a collection tube. The extraction step was repeated until the hexane layer became colorless. The pooled hexane extracts were then evaporated to dryness using a rotary vacuum concentrator (RVC 2-25CD plus, Martin Christ, Osterode am Harz, Germany), and the residue was dissolved in 300 µL of acetonitrile:dichloromethane:methanol (45:20:35, *v*/*v*/*v*) containing 0.1% BHT.

Tocols and retinol were separated and quantified using a SpectraSystem HPLC instrument (Thermo Separation Products, Inc., Waltham, MA, USA) equipped with a quaternary gradient pump, an autosampler, and UV-Vis and FD detectors, following the method of Kurilich and Juvik [31]. Separation was performed using two C18 reversed-phase columns connected in a series: a Vydac 201TP54 column (5 µm, 4.6 × 150 mm; Hichrom, Reading, UK), followed by a Zorbax RX-C18 column (5 µm, 4.6 × 150 mm; Agilent Technologies, Santa Clara, CA, USA). The separation columns were protected by a Supelguard Discovery C18 guard column (5 µm, 4 × 20 mm; Supelco, Bellefonte, PA, USA). The mobile phase consisted of acetonitrile:methanol:dichloromethane (75:25:5, *v*/*v*/*v*) containing 0.1% BHT and 0.05% triethylamine. An aliquot of 30 µL was injected, and the flow rate was 1.8 mL/min. The separations were performed at room temperature. Tocols were detected by fluorescence (excitation 290 nm, emission 330 nm), whereas retinol was monitored by UV-Vis detection at 325 nm. Compound identification was based on retention time matching with standards, and quantification was performed by external calibration using commercially available standards (r^2^ ≥ 0.99). Tocol and retinol standards (retinol; α-, γ-, and δ-tocopherol; and α- and γ-tocotrienol; purity ≥ 93%) were obtained from Supelco (Sigma-Aldrich, St. Louis, MO, USA).

### 2.5. Analysis of Fatty Acid Profile in Egg Yolk

The fatty acid (FA) profile was determined in lipids extracted from egg yolk samples according to the method described by Smedes [32]. Fatty acid composition was analyzed by gas chromatography following conversion of lipids to fatty acid methyl esters (FAMEs) in accordance with ISO Standard [33]. Briefly, 1 μL of the FAME solution was injected into an Agilent 6890N gas chromatograph (Agilent Technologies, Santa Clara, CA, USA) equipped with a flame ionization detector (FID), as specified in ISO Standard [34]. Separation was performed on a DB-23 capillary column (60 m × 0.25 mm i.d. × 0.25 μm film thickness; Agilent Technologies). Helium was used as the carrier gas at a flow rate of 1.5 mL/min. The injector and detector temperatures were set at 250 °C and 280 °C, respectively. The oven temperature was programmed from 60 °C to 220 °C at a rate of 7 °C/min and held at 220 °C for 17 min. The split ratio was 30:1. FAMEs were identified by the comparison of their retention times with those of commercial FAME standards (C8–C22). Results were expressed as a percentage of total fatty acids. In addition, the proportions of saturated fatty acids (SFAs), monounsaturated fatty acids (MUFAs), polyunsaturated fatty acids (PUFAs), and n3 and n6 fatty acids, as well as the n6/n3 ratio, were calculated.

### 2.6. Analysis of Egg Oxidative Stability

#### 2.6.1. Analysis of Oxidative Stability During Storage

In total, 12 eggs were randomly selected from each cage to determine oxidative stability during storage at different temperatures. Three eggs from each cage of each dietary treatment were analyzed directly for yolk malondialdehyde (MDA) content, while the others were stored at 4 °C, room temperature (22 °C), and 30 °C for 2 weeks and analyzed in sets of 3 eggs. At the end of the storage period, 3 eggs from each dietary treatment that had been stored at a specific temperature were broken, and the yolks were combined and mixed with a spatula. The combined yolk samples were used to analyze the MDA content as described by Botsoglou et al. [35]. A 2 g egg yolk sample was weighed in duplicate into a 50 mL PP tube and homogenized using a T10 Ultra-Turaxx (IKA, Staufen, Germany) with 8 mL of a 5% aqueous solution of trichloroacetic acid and 5 mL of hexane containing 0.8% butylated hydroxytoluene. The tubes were centrifuged (4000 rpm, 5 min; Centric 322A, Tehtnica, Železniki, Slovenia) and the upper hexane layer was discarded, while the lower aqueous layer was filtered, if necessary, and used for further analysis. An aliquot of 2.5 mL was pipetted into a 15 mL PP screw cap tube, and 1.5 mL of a 0.8% aqueous solution of 2-thiobarbituric acid was added. After incubation at 70 °C for 30 min, the tubes were cooled, and the absorbance was measured at 532 nm (Helios γ, Thermo Electron Corporation, UK). The MDA content was calculated using a standard calibration curve prepared using 1,1,3,3-tetramethoxypropane as the precursor of MDA.

#### 2.6.2. Analysis of Fe-Induced Lipid Oxidation

The susceptibility of eggs to iron-induced lipid oxidation was assessed following the procedure of Botsoglou et al. [36], using three eggs per cage. The yolks were pooled and thoroughly mixed using a spatula. A 3 g egg sample was weighed in triplicate into 50 mL polypropylene tubes (3 g) and homogenized using T10 Ultra-Turaxx with 27 mL of 1.15% KCl. A 2.5 mL aliquot of the homogenate was then mixed with 12.5 mL of 80 mM TRIS-maleate buffer, pH 7.4; 5 mL of 5 mM FeSO_4_ × 7H_2_O; and 5 mL of 2 mM ascorbic acid. After mixing, samples were incubated at 37 °C for 0, 100, and 200 min. At the indicated times, 2 mL aliquots of each incubate were immediately subjected to the determination of MDA content as described in Section 2.6.1. The results from each cage were used to obtain a linear regression of MDA content over incubation time, and the slope was determined.

### 2.7. Statistical Analysis

The data were analyzed using SAS software (version 9.4; SAS Institute Inc., Cary, NC, USA). The feeding trial was carried out as a completely randomized experiment with a factorial structure including two maize hybrids differing in grain hardness, three vitamin A supplementation levels, and two trace mineral sources (inorganic or organic), resulting in 12 dietary treatments. A cage containing three hens was considered the experimental unit. The normality of the data distribution was tested using the Shapiro–Wilk test. Normality was assumed when the Shapiro–Wilk test was not significant (*p* > 0.05). All properties were normally distributed, and data did not require a transformation. Differences in the fatty acid profile and oxidative stability were analyzed using the MIXED procedure, with corn hybrid, vitamin A level, and trace mineral form as fixed effects, using the following model:

(1) $$Y_{ijkl}=\;\mu +\alpha_{i}+\beta_{j}+\gamma_{k}+{\left(\alpha \beta \right)}_{ij}+{\left(\alpha \gamma \right)}_{ik}+{\left(\beta \gamma \right)}_{jk}+{\left(\alpha \beta \gamma \right)}_{ijk}+\varepsilon_{ijkl}$$

where *Y_ijkl_* is the observation *l* in the level of hybrid, level *j* of vitamin A supplementation level, and level *k* of trace mineral form; *μ* is the overall mean; *α_i_* is the fixed treatment effect of hybrid *i*; *β_j_* is the fixed treatment effect of vitamin A supplementation level *j*; *γ_k_* is the fixed treatment effect of trace mineral form *k*; *(αβ)_ij_* is the effect of the interaction of the hybrid *i* with vitamin A supplementation level *j*; *(αγ)_ik_* is the effect of the interaction of the hybrid *i* with trace mineral form *k*; *(βγ)_jk_* is the effect of the interaction of the vitamin supplementation level *j* with trace mineral form *k*; *(αβγ)_ijk_* is the interaction between hybrid *i*, vitamin A supplementation level *j*, and trace mineral form *k*; and *ε_ijkl_* is the random error. The same procedure was used to analyze differences between treatments in production performance, egg quality parameters, and yolk tocol and retinol contents using repeated measurements ANOVA, with results obtained from the 21st to 63rd day of the trial, using the following model:

(2) $$Y_{ijklt}=\;\mu +\alpha_{i}+\beta_{j}+\gamma_{k}+{\left(\alpha \beta \right)}_{ij}+{\left(\alpha \gamma \right)}_{ik}+{\left(\beta \gamma \right)}_{jk}+{\left(\alpha \beta \gamma \right)}_{ijk}+\delta_{ijkl}+\tau_{t}+{\left(\alpha \tau \right)}_{it}+{\left(\beta \tau \right)}_{jt}+{\left(\gamma \tau \right)}_{kt}\;+{\left(\alpha \beta \tau \right)}_{ijt}+{\left(\alpha \gamma \tau \right)}_{ikt}+{\left(\beta \gamma \tau \right)}_{jkt}+{\left(\alpha \beta \gamma \tau \right)}_{ijkt}+\varepsilon_{ijklt}$$

where *Y_ijklt_* is the observation *l* in the level of hybrid, level *j* of vitamin A supplementation level, and level *k* of trace mineral form in the period of day *τ*; *δ_ijkl_* is the random error (the variance between cages within treatments); *τ_t_* is the effect of day *t*; (*ατ_it_*) is the effect of interaction between hybrid *i* and day *t*; *(βτ_jt_)* is the effect of interaction between vitamin A supplementation level *j* and day *t*; (*γτ_kt_*) is the interaction between trace mineral form *k* and day *t*; (*αβτ*)*_ijt_* is the effect of the interaction between hybrid *i*, vitamin A supplementation level *j*, and day *t*; (*αγτ*)*_ikt_* is the effect of the interaction between hybrid *i*, trace mineral form *k*, and day *t*; (*βγτ*)*_jkt_* is the effect of the interaction between vitamin supplementation level *j*, trace mineral form *k*, and day *t*; (*αβγτ*)*_ijkt_* is the interaction between hybrid *i*, vitamin A supplementation level *j*, trace mineral form *k*, and day *t*; and *ε_ijklt_* is the random error (variance between measurements within cages). Differences among treatments were assessed using Tukey-adjusted comparisons of least-squares means. Mean values were defined by the least squares means statement and compared using the PDIFF option. The relationship between the obtained egg properties was analyzed using the CORR procedure. The threshold for statistical significance was defined as *p* < 0.05.

## 3. Results

### 3.1. Production Performance

The corn hybrid, the vitamin A supplementation level, and the trace mineral form showed different effects on the production performance of the laying hens (Table 3). While diet intake and egg production were not affected by the three tested dietary variables or their interactions, this was the case for egg weight, egg mass, and feed conversion ratio (FCR). Egg weight and mass were affected by the corn hybrid, the vitamin A supplementation level, and their interaction (*p* < 0.05). Since the hybrid × trace mineral form interaction was also found to be significant, the effect of the corn hybrid is dependent on the other dietary components. The vitamin A supplementation level was the only main factor affecting egg weight (*p* < 0.05), with eggs from hens fed the diet with the lowest vitamin A supplementation being approximately 2% lighter. In addition to the main effect of the hybrid, FCR showed an interaction, suggesting hybrid- and mineral-specific responses to vitamin A levels.

### 3.2. Egg Quality Parameters

The hybrid, the vitamin A supplementation level, and the form of the trace mineral influenced the tested parameters of egg quality, with the exception of the shape index, egg surface area, yolk weight, shell thickness, and shell L* (Table 4, Table 5 and Table 6). Egg height and width were affected by trace mineral form and vitamin A supplementation level (*p* < 0.05). Inorganic trace minerals resulted in higher egg height and width, although this increase was small (<1%). The hybrid × vitamin A supplementation level interaction showed that the hard-type hybrid achieved lower height in eggs from hens fed diets supplemented with 5000 IU/kg. This hybrid also achieved a lower egg width in hens fed diets supplemented with 10,000 and 20,000 IU/kg. Similarly, albumen weight was also higher (*p* < 0.01; increase of 3.0 and 1.1%) in eggs from hens fed diets with inorganic trace minerals and higher vitamin A supplementation levels. The soft-type hybrid and the inorganic trace mineral form in diets resulted in higher shell weight (*p* < 0.05).

The internal egg quality parameters, including albumen height, YCF, and Haugh units, were affected by several factors (Table 5). The corn hybrid affected albumen height and Haugh units (*p* < 0.05), with the soft-type hybrid resulting in higher values. The form of trace elements affected albumen height and tended to affect Haugh units (*p* = 0.009 and 0.063, respectively), with the inorganic form leading to higher values. However, this effect depended on the level of vitamin A supplementation. While the values of these parameters increased with increasing levels in diets with inorganic trace minerals, they decreased in diets with organic trace elements. Both vitamin A supplementation level and mineral form had an effect on YCF values (*p* < 0.001), with higher values in eggs from hens fed hard-type hybrids, organic trace minerals, and with the lowest and highest levels of vitamin A supplementation. The vitamin A supplementation level in the laying hen diet increased the shell strength of the eggs (*p* < 0.05), with strength increasing with increasing supplementation level.

Compared to the YCF scale, the more sensitive colorimetric analysis of yolk and shell color in the CIE Lab* space demonstrated that the corn hybrid significantly affected almost all yolk color parameters (Table 6). Laying hens fed diets based on soft-type hybrid resulted in egg yolks of higher brightness and yellowness and lower redness compared to egg yolks of laying hens fed with hard-type hybrid-based diets (*p* < 0.001). In addition, the use of trace minerals in organic form resulted in higher values for yolk yellowness compared to inorganic trace minerals (*p* < 0.05), while lower vitamin A supplementation level resulted in higher values for yolk redness compared to intermediate level (*p* < 0.01). In contrast to yolk color, the hard-type hybrid in the laying hen diet resulted in shells of higher redness and yellowness compared to the soft-type hybrid (*p* < 0.01). On the other hand, eggs from hens fed with inorganic trace minerals were found to have higher shell yellowness values.

### 3.3. Content of Tocols and Retinol in Egg Yolks

**Figure 1:**
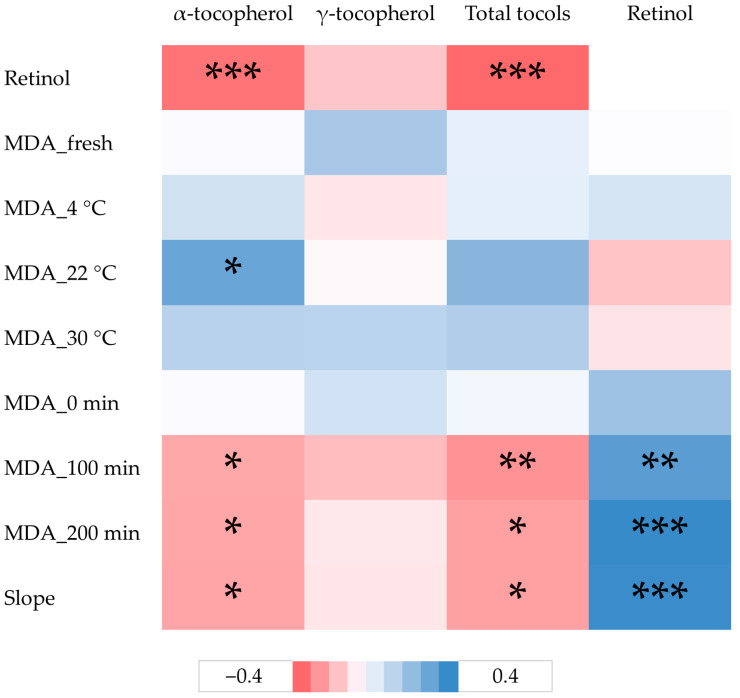
Correlations between yolk tocol and retinol contents and oxidative stability of eggs during storage at different temperatures and under Fe-induced oxidation conditions using yolk emulsions. Malondialdehyde content during storage: MDA_fresh—in fresh eggs, MDA_4 °C—in eggs stored for two weeks at 4 °C, MDA_22 °C—in eggs stored for two weeks at 22 °C, MDA_30 °C—in eggs stored for two weeks at 30 °C. Malondialdehyde content under Fe-induced oxidation conditions: MDA_0 min—in yolk emulsions at the beginning of incubation, MDA_100 min—in yolk emulsions incubated for 100 min, MDA_200 min—in yolk emulsions incubated for 200 min.* *p* = 0.05–0.01; ** *p* = 0.01–0.001; *** *p* < 0.001.

The hybrid influenced the content of individual tocopherols (*p* < 0.001; Table 7), but the total amount of tocols in the yolk remained similar for both tested hybrids. The use of the hard-type corn hybrid in the hen diet resulted in an approximately 5.3% higher content of α-tocopherol and a 7.4% lower content of γ-tocopherol. However, it should be noted that the effect of the hybrid on α-tocopherol content depended on the vitamin A supplementation level and tended to depend on the trace mineral form (*p* = 0.040 and 0.059, respectively). The form of the trace elements only affected the γ-tocopherol content in the yolk, with the content being higher in eggs from hens fed diets containing inorganic trace mineral form. On the other hand, the contents of all tested compounds were also affected by vitamin A supplementation level, but the effect was different for different groups of compounds (*p* < 0.001). The contents of tocols decreased with increasing vitamin A supplementation level; supplementation of 20,000 IU/kg in hen diets resulted in 12.6, 11.1, and 12.1% lower contents of α-tocopherol, γ-tocopherol, and total tocols, respectively, compared to supplementation of 5000 IU/kg. Furthermore, the effect on α-tocopherol and total tocols depended on trace mineral form. In eggs from hens fed diets with organic trace minerals, the highest contents were achieved with the lowest vitamin A supplementation level, while in diets with inorganic trace minerals, the highest contents were achieved at the intermediate vitamin A supplementation level. In contrast to tocols and in agreement with negative correlations (r = −0.355 for α-tocopherol and r = −0.378 for total tocols, *p* < 0.001; Figure 1), the level of retinol in the yolk increased with increasing dietary vitamin A supplementation level, by 13.1% from 5000 to 20,000 IU/kg. Although the vitamin A supplementation level was the most important factor influencing the retinol content in the yolk, feeding the soft-type hybrid to hens tended to result in a higher content than the hard-type hybrid (*p* = 0.070). In agreement with the observed interaction trace mineral form × vitamin A supplementation level, the highest contents of α-tocopherol and total tocols were found in eggs from hens whose diet was supplemented with 5000 IU/kg of vitamin A and organic trace minerals (14.76 and 19.75 µg/g, respectively).

### 3.4. Yolk Fatty Acid Profile

Egg yolks from hens fed the tested dietary treatments showed the highest proportion of *cis*-oleic acid (C18:1; on average 38.7% of total fatty acids), followed by palmitic acid (C16:0, 25.5%), *cis*-linoleic acid (C18:2, 20.3%), and stearic acid (C18:0, 9.0%) (Table 8a,b). With the exception of palmitoleic acid (C16:1, 2.2%) and arachidonic acid (C20:4n6, 2.3%), the proportions of the remaining fatty acids were below 0.4%. The overall proportions of SFAs, MUFAs, and PUFAs were 35.0%, 41.6%, and 23.4%, respectively. The average proportion of n6 fatty acids accounted for 2.7% of total fatty acids, which was 14.2 higher than the proportion of n3 fatty acids.

The fatty acid composition of egg yolk was primarily influenced by the corn hybrid used in the diet (*p* < 0.05), affecting the proportions of nine out of the fifteen identified fatty acids. Consequently, the corn hybrid significantly influenced the total content of SFAs, MUFAs, PUFAs, and n3 fatty acids, as well as the n6/n3 ratio (*p* < 0.05). Diets based on the hard-type corn hybrid resulted in higher proportions of palmitic acid, palmitoleic acid, and *cis*-oleic acid, whereas diets containing the soft-type hybrid led to higher proportions of heptadecanoic acid (C17:0), *cis*-linoleic acid, *γ*-linolenic acid (C18:3n6), *α*-linolenic acid (C18:3n3), and nervonic acid (C24:1). Overall, yolks from hens fed hard-type corn hybrid diets had higher proportions of SFAs and MUFAs, while yolks from hens fed soft-type hybrid diets contained approximately 8% more PUFAs.

The form of trace minerals had a minimal effect on the yolk fatty acid profile, influencing only the proportion of *trans*-oleic acid (*p* < 0.01). In contrast, the level of vitamin A supplementation significantly affected the proportions of *cis*-oleic acid, arachidonic acid, and nervonic acid (*p* < 0.05). For these fatty acids, supplementation levels of 5000 and 20,000 IU/kg resulted in similar proportions, which were higher than the content of *cis*-oleic acid and lower than the contents of arachidonic acid and nervonic acid in eggs from hens fed diets supplemented with 10,000 IU/kg.

### 3.5. Yolk Oxidative Stability

The oxidative stability of eggs from hens fed dietary treatments was determined after the storage of the eggs at different temperatures and under Fe-induced oxidation conditions using yolk emulsions (Table 9). During storage, the MDA content in the yolk showed minor variations depending on the storage temperature. Nevertheless, some significant effects were observed. The hybrids and vitamin A supplementation level affected the MDA content in the yolk of eggs stored at 30 °C for a fortnight, with higher values found in eggs from hens fed diets with hard-type corn hybrids and with the lowest vitamin A supplementation level (*p* < 0.01). In eggs stored at 4 °C, supplementation of 10,000 IU/kg of vitamin A resulted in a 17.8% higher MDA content in the yolk (*p* < 0.05). At the same storage temperature, the addition of trace minerals in organic form led to an 18.2% higher MDA content compared to the inorganic trace minerals (*p* < 0.01). Based on the significant interaction, the effects of vitamin A supplementation level also depended on the trace mineral form. Regardless of the observed effects on some parameters of oxidative stability during storage, MDA contents were minimally associated with yolk tocol and retinol contents (Figure 1).

Under Fe-induced lipid oxidation conditions, the MDA content in the yolks increased linearly with the duration of incubation of the yolk emulsion; after 100 min, the content was 2.3-fold and after 200 min 4-fold higher than at the beginning of the incubation. The increase in MDA content in the yolk was higher in eggs from hens fed diets based on hard-type corn hybrid, resulting in a steeper slope of the curve of MDA content in the yolk over the duration of incubation compared to soft-type hybrid (*p* < 0.05). While trace mineral form had no effect on the MDA formed during Fe-induced lipid oxidation, the level of vitamin A supplementation affected the MDA content after 100 and 200 min of incubation and the resulting slope (*p* < 0.001); however, the response depended on the hybrid. The highest vitamin A supplementation led to the highest MDA content in the yolk and the largest slope of the curve of MDA content in the yolk over the duration of incubation, in agreement with the positive correlation between yolk retinol content and MDA content in yolk emulsions after 100 and 200 min of incubation (Figure 1; r = 0.296 and r = −0.359, respectively, *p* < 0.01). On the other hand, the higher yolk content of α-tocopherol and total tocol resulted in lower MDA content in yolk emulsions (r = −0.251 and r = 0.255, respectively, *p* < 0.05).

## 4. Discussion

The dietary treatments investigated in the present study represent small changes in the diet of laying hens, but with potential impact on production performance and egg properties. These changes included the corn hybrid, the trace mineral form, and the vitamin A supplementation level. The trace minerals were supplemented in the recommended amounts depending on the bioavailability of the organic and inorganic forms, while vitamin A was supplemented in the recommended amount and in doses twice lower and twice higher.

Although the type of corn hybrid had no significant effect on diet intake, egg production, and average egg weight, hens fed the hard-type corn hybrid produced a higher daily egg mass and had a slightly better FCR (Table 3). The effect of corn genotype on some parameters of production performance observed in the present study is consistent with the results obtained by Gunjević et al. [37] on 15 corn hybrids in laying hens and by Melo-Durán et al. [38] on 8 varieties in broilers. The results obtained indicate improved nutrient digestibility and utilization, which is consistent with the studies linking vitreous endosperm and grain hardness to slower starch digestibility [39]. Trace mineral form did not affect production performance of the laying hens, supporting high bioavailability of organic trace minerals, which are added to the diet in lower quantities than inorganic trace minerals. This result was not expected, as the meta-analysis by Byrne et al. [40] showed that the complete replacement of inorganic trace minerals by organic trace minerals leads to a higher egg mass (+0.5 g/hen/day) and a higher egg weight (+0.48 g). However, it should be noted that inorganic trace minerals tended to lead to heavier eggs compared to organic trace minerals in the present study (*p* = 0.095). Therefore, it is possible that, in the present study, organic trace minerals were supplemented in a sufficient quantity not to impair the production performance of laying hens compared to inorganic trace minerals, but not in a sufficient quantity to reach their potential. On the other hand, supplementation of vitamin A at higher levels (10,000–20,000 IU/kg) improved egg weight and mass (Table 3), supporting its known role in improving oviduct secretory function and yolk formation, as shown by Chen et al. [41] for broiler breeders. However, no further improvement in these production performance parameters was observed beyond 10,000 IU/kg as recommended by layer breeders [28,42], suggesting a threshold beyond which additional supplementation may not provide economic benefits.

The effect of trace mineral form and vitamin A supplementation level on egg weight and egg mass depended on the corn hybrid (Table 3). It appears that hybrid-dependent properties of the grain used in laying hen diets can modify the utilization of other dietary nutrients. The physical structure of grain is related to vitreousness and determines hardness [18], which in turn affects grain milling properties. Grain of higher hardness produces larger particles with more irregular shapes that are retained longer in the digestive tract than grain of lower hardness [43]. As a result, this can improve the utilization of all dietary nutrients, as shown for calcium and phosphorus [44], which may also translate into a hybrid-dependent effect on FCR. On the other hand, the lowest weight measured in the present study was found in eggs from hens fed diets based on hard-type hybrid supplemented with 5000 IU/kg of vitamin A, suggesting that vitamin A utilization can be impaired at lower supplementation levels in hybrids with larger milling particles. Furthermore, the effect of vitamin A supplementation level on egg weight depended on the trace mineral form, and higher egg weight was achieved when laying hen diets were supplemented with inorganic rather than organic trace minerals. A possible reason for this could be interactions between vitamin A and zinc, as vitamin A deficiency results in impaired zinc absorption [45], which can affect egg weight and FCR [46].

In the present study, a wide range of egg quality parameters was determined to gain a comprehensive insight into the effects of corn type, trace mineral form, and vitamin A supplementation level. The egg dimensions and egg part weights were not uniformly affected by these factors (Table 4). Although trace mineral form and vitamin A supplementation had no effect on the shape index, they influenced the height and width of the egg. Eggs from hens fed diets containing inorganic trace minerals laid larger eggs, which is consistent with the observed tendency for heavier eggs (Table 3). However, it should be noted that the effect of trace mineral form on egg size also depended on the vitamin A supplementation level, as observed in egg width and egg surface area. A possible reason for this finding could be the previously mentioned vitamin A–zinc interaction, as higher zinc supplementation levels lead to heavier [46], and thus, larger eggs. The larger eggs laid by hens fed inorganic trace minerals were also accompanied by higher egg shell and albumen weights. The meta-analysis by Byrne et al. [40] showed that the complete replacement of inorganic trace minerals by organic trace minerals leads to higher egg shell weight (+0.20 g/egg), which was not found in the present study. The results obtained, therefore, confirm that organic trace minerals were not supplemented in sufficient quantities to have a full effect on egg weight and dimensions. In agreement with the effect on egg weight (Table 3), higher vitamin A supplementation level in the hen diet resulted in larger eggs (higher egg height and width) and higher albumen weight. The effect on egg height was hybrid-dependent, and the lowest height was observed in eggs from hens fed diets with the hard-type hybrid and supplemented with 5000 IU/kg of vitamin A, reflecting the hybrid × vitamin A supplementation level effect observed for egg weight (Table 3). Surprisingly, the corn hybrid had an effect on eggshell weight. The reasons for this effect remain to be clarified; however, it is possible that the mineral composition of the tested hybrids could influence egg shell formation [47].

Although a previous study with 15 corn hybrids showed no effect of hybrid on albumen height and Haugh units [37], this effect was found to be significant in the present study (Table 5). The average albumen height and Haugh units were 7.21 mm and 83.02 for the soft-type hybrid and 6.82 mm and 80.71 for the hard-type hybrid, respectively, which are within the range reported by Gunjević et al. [37] (6.80–7.81 mm and 80.07–88.03, respectively). The two corn hybrids used were selected based on the grain hardness, and it seems that their differences were distinct enough to detect the effects on albumen quality. Since the quality of dietary protein and its availability could affect albumin quality [48], it is possible that the hardness of the hybrids tested could be the reason for this effect. Harder corn hybrids have a higher content of zein [16], which has a low content of some essential amino acids such as lysine and methionine [49]. In addition, zein proteins form a protein–starch matrix that is denser in harder hybrids, which limits the digestibility of corn starch and proteins [15]. The albumen height was also affected by trace mineral form in the tested diets. The effect of the trace mineral form is more associated with the egg shell properties, which was not the case in the present study. However, it is possible that mineral bioavailability differences (especially for zinc) between organic and inorganic forms impaired protein deposition and were reflected in changes in albumen properties [23]. However, the effect depended on the vitamin A supplementation level, and albumen height responded differently to vitamin A supplementation level depending on the trace mineral form, resulting in a decrease with increasing vitamin A supplementation level in diets with organic trace minerals. Since the bioavailability of trace minerals differs based on their form [23], it is possible that the same vitamin A level can result in different functional outcomes depending on the mineral form supporting specific biological functions, as seen in the present study with albumen height and Haugh units. In contrast to the trace mineral form, the level of vitamin A supplementation affected shell strength (Table 5). The increasing shell strength with increasing vitamin A supplementation level is consistent with the positive effect of vitamin A on shell formation [50]. However, lower shell strength can be achieved at lower vitamin A supplementation levels in diets containing organic trace mineral forms. This finding suggests that egg formation is adequate when the shell gland is properly supported, and adequate vitamin A supplementation has been shown to improve the antioxidant status of the shell gland [51].

The significant effects of all three factors investigated in the present study are evident in yolk color (Table 5 and Table 6). The hard-type corn hybrid in the hen diet led to higher YCF scores and greater redness compared to the soft-type corn hybrid, which resulted in paler yolks, as indicated by higher lightness and yellowness values. The effect of the corn hybrid was expected and aligns with its impact on yolk carotenoid content, as yolk color intensity increases with higher carotenoid content in the diet [52]. The YCF score was also influenced by trace mineral form and vitamin A supplementation level, although the differences between main effects were minimal. Organic trace minerals resulted in slightly higher YCF values, accompanied by increased b* values. This effect may be related to the stronger antioxidant support provided by organic forms, even at lower supplementation levels compared to the inorganic form [15], allowing carotenoids to be used less as antioxidants and thus deposited in higher proportions in the egg yolk. In contrast, the effect of the vitamin A supplementation level was not as conclusive as expected. Zurak et al. [52] showed that increased dietary vitamin A reduces the bioavailability of corn carotenoids in laying hens, so a decrease in yolk color with increasing vitamin A supplementation level was expected. Of the tested yolk color parameters, only YCF and a* values were affected by this factor, and the lowest and highest vitamin A supplementation levels in the hen diet resulted in similar values. Zurak et al. [52] found that dietary vitamin A level affected the deposition of only some carotenoids, so it is possible that these differences resulted from different carotenoid profiles in yolks from hens fed diets with varying vitamin A supplementation levels. In addition, the effect on both YCF and a* values depended on the hybrid, consistent with the variation in bioaccessibility of different carotenoids from various corn hybrids [27].

α- and γ-tocopherols were the only tocols found in yolks from hens fed the dietary treatments, while other corn tocols were present at levels too low to be quantifiable in egg yolk (Table 7). As the content of bioactive compounds in yolk can be modified by dietary composition [1], an effect of the hybrid on α- and γ-tocopherol contents, and thus total tocols, was expected, reflecting differences between the two hybrids used in formulating the laying hen diets. This also takes into account that all diets were supplemented with the same amount of vitamin E as α-tocopheryl acetate (Table 2 and Table S1B). The significant interactions between hybrid and both trace mineral form and vitamin A supplementation level suggest that yolk contents of α-tocopherol and total tocols can be further modulated. Compared to inorganic trace minerals, organic trace minerals resulted in higher yolk contents when hens were fed diets based on the hard-type hybrid, while the contents were lower in diets based on the soft-type hybrid. It is possible that longer exposure of digesta in the digestive tract, which could be caused by hard-type hybrids, favors organic trace minerals that do not lead to bile salt precipitation and decreased micellarization of fat-soluble compounds such as tocols [43,53]. The longer exposure in the digestive tract, which allows more time for micellarization and further absorption of fat-soluble compounds, could also be a reason for the smaller decrease in yolk contents of α-tocopherol and total tocols in diets based on hard-type hybrids at the highest vitamin A supplementation level. The hybrid did not affect yolk retinol content (Table 7), suggesting that retinol was influenced only by supplementation and not by the contribution of provitamin A carotenoids in corn. Trace mineral form affected only the yolk content of γ-tocopherol, with slightly higher values in eggs from hens fed the inorganic trace mineral form. Similar findings were not found in the literature, so the reasons for this effect on yolk γ-tocopherol content remain unclear. A possible explanation is that better mineral supply from diets with inorganic trace minerals, as previously observed for egg quality parameters, could enhance antioxidant status through antioxidant enzymes, preserving γ-tocopherol from oxidation and allowing more to deposit in the yolk. In contrast to the hybrid type and trace mineral form, vitamin A supplementation level significantly affected the contents of all tocols and retinol in yolk (*p* < 0.001). Generally, tocol content decreased with increasing dietary vitamin A supplementation, consistent with the antagonistic effect of dietary retinyl acetate on tocopherol deposition in egg yolk previously reported [54,55], and confirmed by the negative correlations shown in Figure 1. Conversely, vitamin A supplementation level led to a dose-dependent increase in yolk retinol content, with the highest value observed at 20,000 IU/kg. This finding confirms previous reports that dietary retinol is efficiently transferred to egg yolk and can be used for functional egg enrichment [1]. In addition, when dietary vitamin A supplementation levels are lower than recommended (i.e., 5000 IU/kg), the use of organic trace minerals can result in higher α-tocopherol and total tocol contents compared to inorganic trace minerals, probably due to the positive effect on micellarization of fat-soluble compounds such as tocols [43,53].

The increase in yolk MUFA and PUFA content represents not only a compositional change but also a clear improvement in the nutritional value of egg yolk fat for human consumption. In a typical hen’s egg, SFAs account for approximately 30–35% of total yolk fatty acids, MUFAs for 40–45%, and PUFAs for 20–25%, with a predominance of n6 over n3 PUFAs [56]. The average proportions of fatty acid groups in eggs from hens fed the experimental diets in the present study fell within these reported ranges. All tested diets were supplemented with 3% sunflower oil, a fat source rich in linoleic acid and practically devoid of α-linolenic acid. This dietary formulation provided a common baseline of high dietary n6 PUFAs and low n3 PUFAs in all treatments, which likely contributed to the relatively high yolk n6 content and elevated n6/n3 ratios observed across eggs from the tested dietary treatments [57]. Consequently, differences in the yolk n6/n3 ratio among treatments are likely driven primarily by variations in n3 fatty acid deposition rather than by substantial changes in n6 supply, potentially reflecting the effects of corn hybrid, vitamin A supplementation level, and trace mineral form on fatty acid metabolism and yolk incorporation.

Dietary treatments modified the yolk fatty acid profile, shifting it toward higher content of certain MUFAs and PUFAs and lower SFA content, depending on the dietary treatment. The fatty acid composition of yolks was influenced predominantly by the corn hybrid used in the diet (Table 8a,b). In general, diets based on hard-type corn increased MUFA content while reducing PUFA content, particularly linoleic and α-linolenic acids, compared with diets containing soft-type corn. As a result, the n6/n3 ratio was significantly less favorable in the treatments with diets based on hard corn (*p* = 0.025). Although the dietary treatments produced relatively small differences in fatty acid profiles (Table 1), the effect of the hybrid is consistent with the literature showing that even small ingredient changes can lead to measurable, though modest, shifts in yolk fatty acids, compared to the larger changes caused by the addition of oils rich in n3 or n6 PUFAs [57]. Trace mineral form had minimal effect on the fatty acid profiles of yolks from eggs produced by hens fed the tested dietary treatments, which aligns with previous findings. Trace minerals are commonly associated with eggshell quality, antioxidant status, and overall production rather than with direct changes in yolk fatty acid composition, unless changes in oxidative protection cause secondary effects [23]. Changes in dietary vitamin A supplementation level resulted in small but statistically significant selective changes in the yolk fatty acid profile. The intermediate supplementation level produced fatty acid proportions that differed significantly from both the low and high supplementation levels, as observed for oleic acid, arachidonic acid, total MUFAs, and total n6 PUFAs (*p* < 0.05). These findings are consistent with the role of vitamin A as a metabolic regulator. Retinoids are known to influence desaturase expression and activity in a dose-dependent and sometimes non-linear manner [58], resulting in a non-linear relationship between vitamin A supplementation level and certain fatty acids, such as arachidonic acid, in tissues.

In this study, yolk oxidative stability was assessed by measuring MDA formation both during egg storage and under a pro-oxidant challenge (Fe-induced oxidation in yolk emulsions; Table 9). The first approach provides information on the shelf life of eggs from hens fed different dietary treatments, while the second approach indicates how dietary treatments enrich egg yolks with antioxidant compounds, i.e., the antioxidant capacity yolks achieve when hens are fed specific diets. The values determined for eggs stored at different temperatures were similar to or even lower than those previously reported [59,60], depending on the dietary treatment, indicating high oxidative stability of yolks in the present study during a 2-week storage period at different temperatures. Overall, storage caused only modest changes in yolk MDA; across storage temperatures, yolk MDA varied within a relatively narrow range, though some significant dietary effects were detected. At 30 °C, eggs from hens fed hard-type corn showed higher yolk MDA than those from hens fed soft-type corn diets, and the lowest supplementation vitamin A level (5000 IU/kg) produced the highest MDA, suggesting poorer stability during warm storage. At 4 °C, however, the pattern differed: 10,000 IU/kg vitamin A increased yolk MDA compared with 5000 and 20,000 IU/kg, and organic minerals increased MDA relative to inorganic minerals. These non-monotonic responses imply that lipid oxidation in yolks during storage is influenced by multiple interacting factors (temperature, matrix, and antioxidant/pro-oxidant balance) and may not correspond perfectly with the more aggressive Fe-induced assay. Greater variations in MDA content in eggs stored at 4 and 30 °C from hens fed diets with inorganic trace minerals than in those fed with organic trace mineral support this observation.

The Fe-induced oxidation caused a strong, linear increase in MDA formation, making it a more sensitive indicator among dietary treatments. In all dietary treatments, MDA content increased with incubation time, reaching a maximum of 156.40 ng/g of yolk. These values were comparable to previously reported results [36,61,62]. However, it is important to emphasize the effect of diet on these results, as the response depends on the addition of bioactive compound sources. Although the differences between dietary treatments in this study were numerically small, the most notable effect was from the vitamin A supplementation level. After 100 and 200 min of incubation, MDA content in yolk emulsions increased with higher vitamin A supplementation level, indicating decreased oxidative stability, although this increase was not significant in dietary treatments with 10,000 IU/kg compared to those with 5000 IU/kg of vitamin A. These results suggest that high vitamin A may have a mild pro-oxidizing effect in egg yolks under Fe-induced conditions, which was confirmed by positive correlations between yolk retinol content and MDA content in yolk emulsions after 100 and 200 min of incubation (Figure 1). This finding was unexpected, as retinol has been shown to have antioxidant action [63]. However, it appears that excessive dietary vitamin A compromises egg yolk oxidative stability. A possible explanation is an antagonism between excessive vitamin A and other components of the antioxidant network, such as vitamin E [54,55]. Decreased α- and γ-tocopherol contents in yolks from hens fed dietary treatments with the highest vitamin A supplementation level compared to lower levels and negative correlations between yolk retinol and tocol content (Figure 1) in the present study (Table 7) support previous findings.

While the effect of trace mineral form on MDA content in yolk emulsions under Fe-induced conditions was not observed, the hybrid did affect MDA formation. Higher contents were found in yolks from hens fed diets based on hard-type corn after 100 and 200 min of incubation, resulting in steeper slopes of MDA content curves over incubation time. Since the two tested corn hybrids resulted in different MUFA and PUFA contents in yolk fat (Table 1), it is expected that these changes would relate to the oxidative stability of egg yolks. However, the results are contradictory—the hard-type corn hybrid resulted in eggs with slightly lower PUFA content but worse Fe-induced oxidative stability, while the soft-type hybrid resulted in eggs with slightly higher PUFAs and n-3, but better stability. Consequently, the oxidative stability of yolks from eggs of hens fed diets based on these two hybrids was not primarily determined by the degree of unsaturation. It is possible, therefore, that differences in Fe-induced oxidative stability are instead affected by the antioxidant–pro-oxidant balance in the yolk, particularly regarding tocols and retinol, as evident by correlations shown in Figure 1. Similar patterns have been reported in n3 PUFA-enriched eggs, where substantial increases in yolk PUFAs and n3 only result in marked rises in TBARS when α-tocopherol supplementation is insufficient or absent, whereas adequate vitamin E largely prevents the expected increase in lipid oxidation despite higher unsaturation [62,64,65]. This observation further confirms that the response to the hybrid effect depends on the trace mineral form in the diet. Compared to inorganic trace minerals, diets based on the hard-type hybrid resulted in yolk emulsions less prone to oxidation, while diets based on the soft-type hybrid resulted in yolk emulsions more prone to oxidation when the diets were supplemented with organic trace minerals. Consistent with the same relationship for α-tocopherol and total tocols, the higher tocol content resulting from longer exposure of digesta in the digestive tract, which could be caused by hard-type hybrids [43,53], leads to better egg yolk stability under Fe-induced oxidation conditions.

When considering egg yolk oxidative stability, it is important to note that corn is also a source of other active substances, such as carotenoids and phenolic acids, which could contribute to yolk antioxidant protection. However, hybrid-associated differences in grain hardness (vitreousness) and related zein content [18] could modify the redox potential of these compounds by altering both the composition (profiles) and the bioaccessibility of endogenous antioxidants. Bound phenolic acids vary across maize cultivars with different hardness and can interact with zein [66,67], potentially modifying their release during digestion. Similarly, carotenoid bioaccessibility from maize is matrix-dependent [19,26], indicating that the physical structure of the endosperm could limit the amount of available carotenoids for deposition into egg yolk. In agreement with the observed hybrid effect on oxidative stability under Fe-induced oxidation, these grain-level effects could further alter yolk antioxidant protection and thereby modulate MDA formation.

## 5. Conclusions

Feeding laying hen diets that varied in corn grain hardness, vitamin A supplementation level, and trace mineral form resulted in significant differences in egg composition and oxidative stability, even though overall performance was only moderately affected. The corn hybrid influenced the yolk fatty acid profile and susceptibility to Fe-induced oxidation more than the trace mineral form, demonstrating that relatively subtle changes in cereal characteristics can lead to measurable changes in egg nutritional quality and stability. Vitamin A primarily acted as a modulator of antioxidant status rather than as a classic antioxidant. Increasing dietary vitamin A raised yolk retinol but simultaneously reduced α- and γ-tocopherols and total tocols, and the highest level tested clearly impaired Fe-induced oxidative stability without substantial changes in yolk PUFAs. These results, along with previous reports, indicate that excessive vitamin A can compromise the oxidative protection of egg lipids if not accompanied by adequate vitamin E and other antioxidants. Trace mineral form had comparatively small main effects, although some interactions with corn hybrid and vitamin A suggest that trace mineral source may modulate antioxidant responses within a given diet matrix. The results show that the oxidative stability of eggs is driven more by the balance between pro-oxidant factors and yolk antioxidants than by modest differences in SFAs, MUFAs, and PUFAs alone.

Overall, the results suggest that optimized dietary strategies combining soft-type corn hybrids, moderate levels of vitamin A (up to 10,000 IU/kg), and organic trace minerals may provide a balanced approach to enhance egg quality and nutritional value without compromising shelf life. Future research should explore the mechanisms of nutrient interactions, particularly those affecting antioxidant systems, to further refine laying hen nutrition and functional egg production.

## Figures and Tables

**Table 1:** Fatty acid profile of experimental diets.

Hybrid	Mineral Form	Vitamin A Level	C16:0	C16:1	C18:0	C18:1 *cis*	C18:2 *cis*	C18:3n3	C20:0	C20:1	C22:0	C22:2
		IJ/kg	% of Total Fat									
Soft	Inorganic	5000	9.19	0.131	3.02	28.75	56.90	0.696	0.326	0.202	0.497	0.294
		10,000	9.15	0.126	3.00	28.38	57.34	0.774	0.338	0.201	0.505	0.181
		20,000	8.93	0.126	3.09	28.63	57.31	0.724	0.336	0.201	0.544	0.112
	Organic	5000	9.13	0.126	3.13	28.75	56.91	0.701	0.338	0.198	0.546	0.165
		10,000	9.34	0.126	3.11	28.73	56.71	0.747	0.347	0.214	0.541	0.129
		20,000	9.37	0.132	3.01	28.42	57.24	0.721	0.315	0.191	0.451	0.153
Hard	Inorganic	5000	9.24	0.149	2.97	28.51	57.16	0.778	0.343	0.209	0.490	0.150
		10,000	8.93	0.149	3.09	28.86	57.01	0.740	0.344	0.206	0.554	0.111
		20,000	9.20	0.147	2.96	28.48	57.25	0.764	0.338	0.204	0.463	0.191
	Organic	5000	9.21	0.147	3.00	28.54	57.07	0.790	0.345	0.211	0.501	0.176
		10,000	9.07	0.151	3.05	28.52	57.20	0.783	0.351	0.211	0.535	0.127
		20,000	9.10	0.148	3.03	28.63	57.13	0.776	0.353	0.211	0.519	0.112

**Table 2:** Content of tocols in experimental diets.

Hybrid	Mineral Form	Vitamin A Level	α- Tocopherol	γ- Tocopherol	δ- Tocopherol	α- Tocotrienol	γ- Tocotrienol	Total Tocols
		IJ/kg	µg/g DM					
Soft	Inorganic	5000	22.53	17.66	1.03	0.51	1.82	55.58
		10,000	22.89	16.59	1.15	0.52	1.61	53.75
		20,000	22.22	15.85	1.17	0.50	1.68	52.95
	Organic	5000	22.31	19.47	1.33	0.57	1.75	55.87
		10,000	23.00	18.26	1.19	0.55	1.94	57.31
		20,000	23.50	17.00	1.09	0.61	1.72	56.70
Hard	Inorganic	5000	21.44	23.66	1.35	0.60	2.52	60.78
		10,000	22.18	24.85	1.23	0.70	2.48	62.82
		20,000	22.60	25.86	1.10	0.62	2.39	64.61
	Organic	5000	22.78	24.33	1.08	0.69	2.61	63.05
		10,000	22.84	23.83	1.48	0.63	2.52	62.44
		20,000	21.88	24.39	0.98	0.67	2.42	62.56

**Table 3:** Analysis of variance for production performance parameters with means for the main effect of the investigated factors ^1^.

Source of Variation	Diet Intake	Egg Production	Egg Weight	Egg Mass	FCR ^2^
Hybrid (H)	0.534	0.244	0.670	0.045	0.027
Mineral form (MF)	0.069	0.450	0.095	0.544	0.198
Vitamin A (VitA)	0.834	0.122	<0.001	0.011	0.212
H × MF	0.714	0.657	0.005	0.017	0.190
H × VitA	0.237	0.759	0.019	0.040	0.017
MF × VitA	0.111	0.554	<0.001	0.467	0.003
H × VitA × MF	0.303	0.135	0.473	0.027	0.279
	g	%	g	g	
Hybrid ^3^					
Soft	113.04	96.5	60.44	57.99 b	1.95 a
Hard	112.56	97.4	60.56	58.95 a	1.91 b
Mineral form ^3^					
Inorganic	113.51	96.7	60.75	58.62	1.92
Organic	112.08	97.2	60.25	58.32	1.94
Vitamin A ^3^					
5000 IU/kg	112.82	96.5	59.75 b	57.51 b	1.95
10,000 IU/kg	112.50	96.4	61.18 a	58.56 ab	1.92
20,000 IU/kg	113.08	98.0	60.57 a	59.35 a	1.93

^1^ n = 49 (7 replicates per treatment × 7 weeks of sampling period). ^2^ FCR—feed conversion ratio. ^3^ Means followed by the same letter in the same column do not differ statistically among themselves according to the Tukey test (*p* = 0.05).

**Table 4:** Analysis of variance for egg dimensions and weights of egg parts with means for the main effect of the investigated factors ^1^.

Source of Variation	Height	Width	Shape Index	Egg Surface Area	Weight		
					Shell	Yolk	Albumen
Hybrid (H)	0.079	0.859	0.083	0.299	0.019	0.879	0.961
Mineral form (MF)	<0.001	<0.001	0.618	0.277	<0.001	0.823	<0.001
Vitamin A (VitA)	0.038	<0.001	0.251	0.886	0.067	0.075	0.021
H × MF	0.514	0.649	0.816	0.774	0.329	0.087	0.809
H × VitA	0.042	0.891	0.010	0.292	<0.001	0.515	0.944
MF × VitA	0.085	0.029	0.495	0.033	0.687	0.487	0.022
H × VitA × MF	0.053	0.674	0.190	0.145	0.033	0.461	0.433
	mm	mm	%	cm^2^	g	g	g
Hybrid ^2^							
Soft	55.21	44.16	80.00	149.49	8.01 a	14.33	38.17
Hard	55.03	44.14	88.24	141.55	7.91 b	14.34	38.18
Mineral form ^2^							
Inorganic	55.32 a	44.29 a	80.08	140.46	8.06 a	14.33	38.73 a
Organic	54.92 b	44.01 b	80.15	141.57	7.87 b	14.35	37.62 b
Vitamin A ^2^							
5000 IU/kg	54.96 b	43.95 b	79.98	141.34	7.90	14.19	37.64 b
10,000 IU/kg	55.11 ab	44.22 a	80.26	140.99	7.95	14.27	38.40 a
20,000 IU/kg	55.29 a	44.29 a	80.12	140.73	8.03	14.54	38.49 a

^1^ n = 49 (7 replicates per treatment × 7 weeks of sampling period). ^2^ Means followed by the same letter in the same column do not differ statistically among themselves according to the Tukey test (*p* = 0.05).

**Table 5:** Analysis of variance for egg quality parameters with means for the main effect of the investigated factors ^1^.

Source of Variation	Albumen Height	YCF ^2^ Color	Haugh Units	Shell Strength	Shell Thickness
Hybrid (H)	<0.001	<0.001	0.002	0.894	0.280
Mineral form (MF)	0.009	0.029	0.063	0.128	0.338
Vitamin A (VitA)	0.337	<0.001	0.294	0.014	0.290
H × MF	0.372	0.329	0.451	0.928	0.325
H × VitA	0.368	0.006	0.645	0.809	0.410
MF × VitA	0.008	0.006	0.038	<0.001	0.383
H × VitA × MF	0.743	0.061	0.897	0.426	0.329
	mm			kg cm^−2^	mm
Hybrid ^3^					
Soft	7.21 a	6.54 b	83.02 a	5.25	0.361
Hard	6.82 b	6.80 a	80.71 b	5.26	0.409
Mineral form ^3^					
Inorganic	7.15 a	6.63 b	82.56	5.30	0.363
Organic	6.87 b	6.71 a	81.17	5.22	0.406
Vitamin A ^3^					
5000 IU/kg	6.98	6.69 a	81.78	5.17 b	0.362
10,000 IU/kg	7.12	6.56 b	82.62	5.26 ab	0.357
20,000 IU/kg	6.93	6.76 a	81.20	5.35 a	0.434

^1^ n = 49 (7 replicates per treatment × 7 weeks of sampling period). ^2^ YCF—yolk color fan. ^3^ Means followed by the same letter in the same column do not differ statistically among themselves according to the Tukey test (*p* = 0.05).

**Table 6:** Analysis of variance for egg color determined according to the CIE L*a*b* with means for the main effect of the investigated factors ^1,2^.

Source of Variation	Yolk			Shell		
	L*	a*	b*	L*	a*	b*
Hybrid (H)	<0.001	<0.001	<0.001	0.139	0.008	0.001
Mineral form (MF)	0.268	0.354	0.012	0.355	0.885	0.016
Vitamin A (VitA)	0.058	0.004	0.127	0.479	0.526	0.478
H × MF	0.003	0.205	0.398	0.271	0.559	0.031
H × VitA	0.701	0.002	0.298	0.276	0.172	0.484
MF × VitA	0.280	0.893	0.331	0.028	0.002	0.232
H × VitA × MF	0.191	0.005	<0.001	0.319	0.944	0.103
						
Hybrid ^3^						
Soft	72.47 a	8.97 b	75.54 a	56.57	19.52 b	30.04 b
Hard	71.43 b	10.21 a	72.95 b	55.67	20.39 a	30.86 a
Mineral form ^3^						
Inorganic	72.03	9.64	73.95 b	56.41	19.97	30.74 a
Organic	71.87	9.54	74.53 a	55.84	19.93	30.15 b
Vitamin A ^3^						
5000 IU/kg	71.72	9.82 a	73.91	55.65	20.06	30.59
10,000 IU/kg	72.16	9.35 b	74.38	56.18	20.10	30.52
20,000 IU/kg	71.97	9.59 ab	74.44	56.56	19.70	30.25

^1^ n = 49 (7 replicates per treatment × 7 weeks of sampling period). ^2^ L*—lightness, a*—redness, b*—yellowness. ^3^ Means followed by the same letter in the same column do not differ statistically among themselves according to the Tukey test (*p* = 0.05).

**Table 7:** Analysis of variance for contents of tocols and retinol in egg yolk with means for the main effect of the investigated factors ^1^.

Source of Variation	α-Tocopherol	γ-Tocopherol	Total Tocols	Retinol
Hybrid (H)	0.003	<0.001	0.244	0.070
Mineral form (MF)	0.704	0.010	0.540	0.994
Vitamin A (VitA)	<0.001	<0.001	<0.001	<0.001
H × MF	0.059	0.286	0.057	0.839
H × VitA	0.040	0.280	0.045	0.885
MF × VitA	0.003	0.120	0.002	0.965
H × VitA × MF	0.539	0.232	0.526	0.646
				
Hybrid ^2^				
Soft	13.13 b	4.89 a	18.03	15.26
Hard	13.84 a	4.53 b	18.37	14.96
Mineral form ^2^				
Inorganic	13.43	4.85 a	18.29	15.11
Organic	13.53	4.57 b	18.l1	15.11
Vitamin A ^2^				
5000 IU/kg	14.17 a	4.99 a	19.15 a	14.35 c
10,000 IU/kg	13.70 a	4.65 b	18.35 b	14.75 b
20,000 IU/kg	12.58 b	4.49 b	17.08 c	16.23 a

^1^ n = 49 (7 replicates per treatment × 7 weeks of sampling period). ^2^ Means followed by the same letter in the same column do not differ statistically among themselves according to the Tukey test (*p* = 0.05).

**Table 8:** Analysis of variance for egg yolk fatty acid profile with means for the main effect of investigated factors ^1–3^.

**(a) Analysis of variance for egg yolk fatty acid profile with means for the main effect of the investigated factors ^1^**											
**Source of Variation**		**C14:0**	**C16:0**	**C16:1**	**C17:0**	**C18:0**	**C18:1** ***trans***	**C18:1 *cis***	**C18:2 *cis***	**C18:3n6**	**C18:3n3**
Hybrid (H)		0.028	<0.001	0.004	0.004	0.080	0.474	0.023	<0.001	<0.001	<0.001
Mineral form (MF)		0.174	0.404	0.268	0.778	0194	0.004	0.785	0.207	1.000	0.410
Vitamin A (VitA)		0.335	0.370	0.094	0.079	0.751	0.123	0.028	0.210	0.086	0.246
H × MF		0.099	0.034	0.002	0.099	0.976	0.237	0.468	0.155	0.274	0.410
H × VitA		0.947	0.329	0.057	0.178	0.891	0.309	0.928	0.864	0.872	0.513
MF × VitA		0.711	0.159	0.007	0.246	0.364	0.135	0.309	0.250	0.839	0.395
H × VitA × MF		0.374	0.100	0.635	0.093	0.236	0.101	0.970	0.323	0.065	0.048
		% of total fat content									
Hybrid ^3^											
Soft		0.30 b	25.17 b	2.13 b	0.158 a	8.89	0.112	35.35 b	21.16 a	0.162 a	0.203 a
Hard		0.32 a	25.85 a	2.26 a	0.146 b	9.15	0.110	39.13 a	19.44 b	0.147 b	0.179 b
Mineral form ^3^											
Inorganic		0.32	25.45	2.17	0.153	8.93	0.107 b	38.70	20.53	0.154	0.193
Organic		0.31	25.57	2.21	0.152	9.13	0.114 a	38.78	20.06	0.154	0.188
Vitamin A ^3^											
5000 IU/kg		0.30	25.35	2.13	0.157	8.95	0.108	38.96 a	20.49	0.153	0.192
10,000 IU/kg		0.31	25.61	2.20	0.154	9.08	0.110	38.10 b	20.58	0.161	0.197
20,000 IU/kg		0.32	25.56	2.24	0.146	9.04	0.114	39.16 a	19.83	0.150	0.184
										
**(b) Analysis of variance for egg yolk fatty acid profile with means for the main effect of the investigated factors ^1,2^**											
**Source of variation**	**C20:1**	**C20:2**	**C20:3n6**	**C20:4n6**	**C24:1**	**SFA**	**MUFA**	**PUFA**	**n6**	**n3**	**n6/n3**
Hybrid (H)	0.123	0.088	0.199	0.584	0.005	<0.001	0.011	<0.001	0.398	<0.001	0.025
Mineral form (MF)	0.903	0.586	0.199	0.644	0.629	0.157	0.655	0.263	0.584	0.410	0.536
Vitamin A (VitA)	0.752	0.227	0.592	0.024	0.010	0.267	0.034	0.143	0.024	0.246	0.475
H × MF	0.188	0.055	0.275	0.539	0.109	0.249	0.275	0.139	0.552	0.410	0.844
H × VitA	0.026	0.019	0.026	0.539	0.613	0.717	0.902	0.834	0.558	0.513	0.494
MF × VitA	0.411	0.491	0.332	0.220	0.361	0.342	0.170	0.306	0.212	0.395	0.699
H × VitA × MF	0.155	0.146	0.468	0.688	0.991	0.049	0.991	0.401	0.747	0.048	0.135
	% of total fat content										
Hybrid ^3^											
Soft	0.218	0.243	0.211	2.34	0.354 a	34.53 b	41.16 b	24.31 a	2.71	0.203 a	13.51 b
Hard	0.226	0.234	0.203	2.30	0.319 b	35.46 a	42.04 a	22.50 b	2.65	0.179 b	14.99 a
Mineral form ^3^											
Inorganic	0.222	0.240	0.203	2.31	0.334	34.84	41.53	23.63	2.66	0.193	14.05
Organic	0.222	0.237	0.211	2.34	0.339	35.14	41.67	23.18	2.70	0.188	14.44
Vitamin A ^3^											
5000 IU/kg	0.224	0.238	0.204	2.24 b	0.322 b	34.76	41.74 ab	23.50	2.59 b	0.192	13.71
10,000 IU/kg	0.220	0.244	0.211	2.45 a	0.363 a	35.16	41.00 b	23.84	2.82 a	0.197	14.56
20,000 IU/kg	0.222	0.234	0.206	2.28 b	0.325 b	35.07	42.06 a	22.87	2.63 b	0.184	14.47

^1^ n = 7. ^2^ SFA—saturated fatty acids, MUFA—monounsaturated fatty acids, PUFA—polyunsaturated fatty acids. ^3^ Means followed by the same letter in the same column do not differ statistically among themselves according to the Tukey test (*p* = 0.05).

**Table 9:** Analysis of variance for oxidative stability of eggs with means for the main effect of the investigated factors ^1^.

Source of Variation	During Storage				Fe-Induced Lipid Oxidation			
	Fresh	4 °C	22 °C	30 °C	0 min	100 min	200 min	Slope
Hybrid (H)	0.412	0.697	0.215	0.003	0.043	0.021	0.042	0.028
Mineral form (MF)	0.322	0.004	0.491	0.276	0.822	0.418	0.088	0.853
Vitamin A (VitA)	0.404	0.022	0.074	0.003	0.921	<0.001	<0.001	<0.001
H × MF	0.235	0.34	0.506	0.763	0.395	0.011	0.023	0.021
H × VitA	0.368	0.191	0.128	0.601	0.223	0.125	0.140	0.134
MF × VitA	0.413	0.063	0.739	<0.001	0.470	0.359	0.994	0.999
H × VitA × MF	0.436	0.486	0.049	0.780	0.833	0.898	0.219	0.210
	ng MDA/g egg yolk ^2^							
Hybrid ^3^								
Soft	35.62	31.95	28.45	32.14 b	31.51 a	64.48 b	111.64 b	0.400 b
Hard	29.93	31.26	30.90	36.79 a	29.35 b	76.37 a	134.76 a	0.527 a
Mineral form ^3^								
Inorganic	36.21	28.98 b	30.36	33.64	30.31	72.50	124.07	0.469
Organic	29.33	34.24 a	29.00	35.29	30.55	68.35	122.33	0.458
Vitamin A ^3^								
5000 IU/kg	39.29	29.93 b	32.89	38.25 a	30.49	59.46 b	98.75 b	0.341 b
10,000 IU/kg	28.54	35.14 a	28.18	32.32 b	30.15	67.83 b	114.45 b	0.421 b
20,000 IU/kg	30.50	29.75 b	27.96	32.82 b	30.66	83.99 a	156.40 a	0.629 a

^1^ n = 7. ^2^ MDA—malondialdehyde. ^3^ Means followed by the same letter in the same column do not differ statistically among themselves according to the Tukey test (*p* = 0.05).

## Data Availability

The raw data supporting the conclusions of this article will be made available by the authors upon request.

## Supplementary Materials

The following supporting information can be downloaded at: https://www.mdpi.com/article/10.3390/foods15040692/s1, Table S1A: Ingredient and calculated nutrient composition of the basal diet.; Table S1B: Premixes prepared for nutritional treatments.
