# Supplementary material for: Production Performance and Properties of Eggs from Hens Fed Diets Differing in Corn Grain Hardness, Vitamin A Supplementation Level, and Mineral Form
Source: Foods. 2026 Feb 13;15(4):692. doi: 10.3390/foods15040692 (PMC12940071; doi:10.3390/foods15040692)
Supplement: Supplementary file 1 [file foods-15-00692-s001.zip › Supplementary Tables.pdf]

Supplementary Tables

Table S1A. Ingredient and calculated nutrient composition of the basal diet.

**Ingredient**	**Content (%)**
Maize hybrid	60
Soybean meal	26.2
Sunflower oil	3
Calcium carbonate	8.8
Monocalcium phosphate	1.2
Sodium chloride	0.4
DL methionine	0.15
Vitamin premix	0.25
**Calculated nutrient composition**[1]	
Crude ash	12.52
Crude protein	16.43
Crude fat	5.61
Crude fibre	2.81
Calcium	3.72
Phosphorus	0.60
Phosphorus, available	0.43
Calcium/Phosphorus	6.24
Sodium	0.18
Lysine	0.87
Methionine	0.41
Methionine + cysteine	0.86
Tryptophan	0.18
Threonine	0.62
Starch	38.70
Metabolizable energy (MJ/kg)	11.63

[1]The calculated nutrient composition of the diets was calculated on the basis of the table values for the composition of the feeds used for diets.

Table S1B. Premixes prepared for nutritional treatments. [1]

	Dietary treatment					
Trace minerals	Inorganic			Organic		
Vitamin A, IU	5,000	10,000	20,000	5,000	10,000	20,000
Vitamin D, IU	2,500					
Vitamin E, mg	20					
Vitamin K, mg	3					
Vitamin B1, mg	1					
Vitamin B2, mg	4					
Vitamin B6, mg	3					
Vitamin B12, mg	25					
Vitamin B5, mg	10					
Vitamin B3, mg	30					
Vitamin B9, mg	0,5					
Vitamin B7, µg	50					
Choline, mg	40					
Zn, mg	80	80	80	30	30	30
Mn, mg	80	80	80	30	30	30
Cu, mg	10	10	10	5	5	5
Fe, mg	10	10	10	5	5	5
Se, mg	0.4	0.4	0.4	0.2	0.2	0.2
I, mg	1					

[1] The premixes have been formulated so that 1 kg of feed contains the amounts of vitamins and microminerals indicated in the table.
